# Clinical, Electrodiagnostic, and Ultrasound Findings in 87 Patients With Finger Drop

**DOI:** 10.7759/cureus.57913

**Published:** 2024-04-09

**Authors:** Lisa B Shields, Vasudeva G Iyer, Yi Ping Zhang, Christopher B Shields

**Affiliations:** 1 Neurological Surgery, Norton Neuroscience Institute, Norton Healthcare, Louisville, USA; 2 Neurology/Clinical Neurophysiology, Neurodiagnostic Center of Louisville, Louisville, USA

**Keywords:** neurosurgery, ultrasound, neuromuscular, parsonage-turner syndrome, electrodiagnostic studies, posterior interosseous nerve, finger drop, neurology

## Abstract

Background: The inability to extend the fingers at the metacarpophalangeal and interphalangeal joints leads to finger drop. While wrist drop and foot drop are well recognized, the causes of finger drop are poorly understood.

Aims: This study describes the clinical, electrodiagnostic (EDX), and ultrasound (US) features in patients with finger drop.

Materials and methods: This is a retrospective study of 87 patients presenting with finger drop and referred for EDX studies during the past 10 years. We analyzed the clinical picture, EDX data, and US findings. The patients were categorized into global (all five digits) or partial (limited to 1-4 digits) finger drop.

Results: Fifty-six (64%) patients had global finger drop, while 31 (36%) had partial finger drop. The frequent cause of finger drop was Parsonage-Turner syndrome (PTS) (29 [33%]), followed by trauma (23 [26%]), cervical radiculopathy (16 [18%]), extensor tendon rupture (four [4%]), and compression/entrapment (two [2%]). In 13 (15%) patients, no cause was identified. A total of 13/16 (81%) patients with cervical radiculopathy and four of the patients with tendon rupture had partial finger drop, while 52/64 (81%) with posterior interosseous nerve (PIN) neuropathy had global finger drop. Of the 16 patients who experienced cervical radiculopathy as the cause of the finger drop, 15 patients had C7 and C8 radiculopathy and one patient had C7 radiculopathy. EDX studies of patients with PTS revealed partial axon loss in 18 (62%) patients, conduction block in eight (28%), and total axon loss in four (14%). Enlarged fascicles were observed by US in 40% of patients with PTS. EDX studies of patients who sustained iatrogenic nerve injury causing finger drop demonstrated total axon loss in six (46%) patients, partial axon loss in four (31%), demyelination in two (15%), and conduction block in two (15%).

Conclusions: PIN neuropathy is the most common cause of finger drop, however, lesser-known causes such as cervical radiculopathy and extensor tendon rupture should also be considered. Global finger drop is suggestive of PIN neuropathy, while partial finger drop occurs more often in cervical radiculopathy and tendon rupture. EDX and US studies provide valuable information for localizing the lesion site and may reveal the cause of the finger drop.

## Introduction

Finger drop is characterized by severe weakness of the finger extensors at the metacarpophalangeal (MP) and interphalangeal (IP) joints with preserved strength of the finger flexors, wrist extensors, and wrist flexors [1-4]. Finger extension at the MP joints is controlled by the extensor digitorum communis which originates from the lateral epicondyle of the humerus and passes down the posterior compartment of the forearm [4]. The extensor digitorum communis provides distinct tendons to all four fingers, and the extensor digiti minimi and extensor indicis provide additional tendons to the small and index fingers, respectively. The extensor pollicis longus causes the extension of the thumb at the IP joint, while the extensor pollicis brevis extends the MP and the carpometacarpal joints. Containing only motor fibers, the posterior interosseous branch of the radial nerve supplies all these muscles.

Several conditions may lead to finger drop, including posterior interosseous nerve (PIN) palsy [2,4-7], cervical foraminal stenosis [2,8,9], Guillain-Barré syndrome [1-3,6,10,11], radial nerve palsy [9], multifocal motor neuropathy with conduction block [2,4], amyotrophic lateral sclerosis [4,6], syringomyelia [4,6], myasthenia gravis [2,4,6,12], cortical hand [4], distal myopathies [4,6], ruptured extensor tendon [4], extensor tendon subluxation [4], and trigger finger [4]. Finger drop may be partial with the weakness limited to extensors of certain digits or global with all digits affected [9,10,13]. Selective vulnerability of specific fascicles to injury determines which of the digits are affected. Cervical cord disease or lesions of cervical roots may cause selective weakness of the fourth and fifth finger extensors that results in pseudoulnar clawing [8,9]. Three types of finger drop have been described by Furukawa et al. based on the digits affected and correlating with the cervical nerve roots involved [9,13]. Knowledge of the details of muscle innervation and the varied finger drop patterns permits clinical differentiation between peripheral nerve and cervical root lesions [9]. Partial finger drop may also be the sequelae of forearm trauma with ensuing weakness of extension of the finger(s) depending on the specific branch of the PIN [14] or tendons injured.

We report 87 patients with finger drop who were referred for electrodiagnostic (EDX) studies. The clinical and EDX findings as well as ultrasound (US) features of these patients are highlighted and the value of those studies in the evaluation of patients with finger drop is discussed.

## Materials and methods

Study population and EDX/US studies

**Figure 1:**
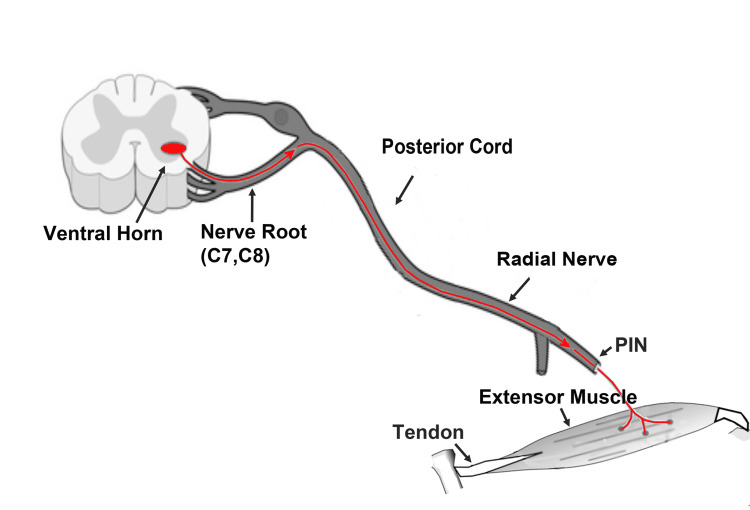
Potential Sites of Lesions That Can Cause Finger Drop The red color represents the course of motor fibers. PIN: posterior interosseous nerve. This figure is the original work of the authors.

This was a retrospective study under an Institutional Review Board (IRB)-approved protocol. Inclusion criteria included patients referred to our facility for EDX studies during a 10-year (2014-2023) period for evaluation of upper extremity weakness, who on clinical examination showed finger drop due to selective weakness of extension of digits. Figure 1 depicts the potential sites of lesions that can cause finger drop. Exclusion criteria included the additional presence of wrist drop or significant weakness of muscles innervated by the median and/or ulnar nerves.

**Figure 2:**
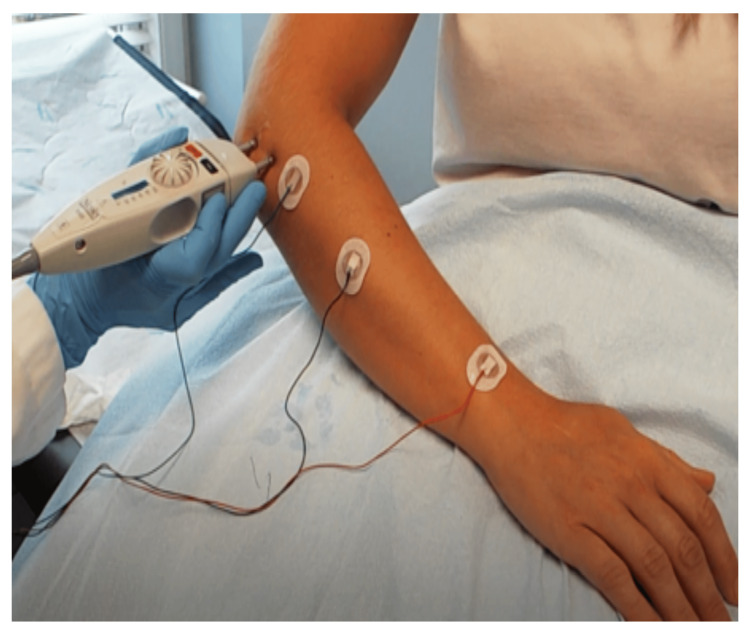
Setup for a Posterior Interosseous Nerve Study Stimulate distal to the lateral epicondyle and record over the extensor digitorum communis or extensor indicis muscles.

Patients underwent clinical neurological examination followed by nerve conduction and needle electromyography (EMG) studies. US evaluation of the radial nerve and its branches was also performed. The EDX studies were performed in our American Association of Neuromuscular and Electrodiagnostic Medicine (AANEM)-accredited facility using a standard protocol of our laboratory [15]. The protocol included motor and sensory conduction studies of the radial, median, and ulnar nerves. Needle EMG was performed using a monopolar needle electrode; muscles innervated by the radial, median, and ulnar nerves were evaluated. The goals were to differentiate between neurogenic finger drop from non-neurogenic causes like tendon rupture and to gain insight into the underlying process (demyelination, conduction block, and partial/total axon loss) as well as accurately localize the site of involvement (Figure 2).

The US studies were conducted using the GE LOGIQ™ E system (GE HealthCare, Chicago, USA) and a 12-18 MHz probe. Short-axis and long-axis views were obtained, and estimation of the cross-sectional area (CSA), alterations in fascicular morphology, and echogenicity were noted.

Several characteristics were collected including patients’ gender and age, laterality (left/right), digits involved, and EDX and US findings.

Ethical approval and informed consent

Informed consent was obtained from all patients. The IRB determined that our study was exempt according to 45 CFR 46.101(b) under Category 4. The IRB number is 22.1021.

## Results

Clinical findings

A total of 87 patients were diagnosed with finger drop based on clinical evaluation (Table 1). The mean age was 56.7 years (range: 17-85 years), and 55 (63%) patients were male. The left side was more commonly (44 [51%] patients) affected. The onset was acute in 69 (79%) patients, chronic in 10 (11%), and unknown in eight (9%). A total of 56 (64%) patients had global finger drop (all digits affected), while 31 (36%) had partial finger drop (1-4 digits affected).

**Table 1:** Demographics, Clinical Features, and Causes of Finger Drop R: right; L: left; A/C/U: acute/chronic/unknown; PTS: Parsonage-Turner syndrome; Iatro: iatrogenic; Inj: injury; Finger drop: G Global, P Partial

Patient Number	Age (years)	Gender (M/F)	Side (R/L)	Onset A/C/U	Partial/Global (P [Digits]/G)	PIN Involved (Y/N)	Cause
1	40	M	L	A	G	Y	Stab injury to forearm
2	51	F	L	A	P 3,4,5	N	C7 radiculopathy
3	63	F	L	A	G	Y	PTS: post-COVID vaccine
4	66	F	L	A	P 1,2,3	Y	Iatro injury: excision of lipoma
5	37	F	L	A	P 4,5	Y	Iatro injury: radial tunnel release
6	79	F	R	C	P 4,5	N	Tendon rupture
7	60	F	L	A	G	Y	Iatro: biceps tendon repair
8	42	M	R	A	G	Y	PTS: heavy lifting
9	85	F	L	A	G	Y	PTS: post-COVID vaccine
10	55	M	R	A	G	N	C7 and C8 radiculopathy
11	54	F	L	C	P 2	Y	Idiopathic
12	35	M	L	A	G	Y	Inj: gunshot wound
13	65	M	R	U	P 3,4,5	N	C7 and C8 radiculopathy
14	29	M	L	A	G	Y	Iatro: repair fracture of radius
15	35	F	L	A	G	Y	Idiopathic
16	45	M	R	A	G	Y	Iatro: biceps tendon repair
17	57	M	R	A	G	Y	PTS: after playing golf
18	68	F	R	C	G	Y	Idiopathic
19	57	M	L	A	G	Y	PTS: post-surgery
20	47	M	R	A	G	Y	PTS: heavy lifting
21	39	M	L	A	G	Y	PTS: post-infection
22	39	M	R	A	G	Y	Iatro: biceps tendon repair
23	59	F	L	U	G	Y	Idiopathic
24	48	F	L	A	G	Y	PTS: no antecedent event
25	72	M	R	A	G	Y	PTS: no antecedent event
26	57	M	L	A	G	Y	PTS: post-infection
27	77	M	R	A	G	Y	PTS: post-infection
28	72	F	L	A	P 3,4,5	Y	PTS: post-surgery
29	55	M	R	A	P 3	N	Iatro: elbow surgery (muscle/tendon injury)
30	52	M	L	A	G	Y	PTS: no antecedent event
31	60	M	L	A	G	Y	Iatro: biceps tendon repair
32	64	F	R	A	G	Y	PTS: post-surgery
33	54	F	L	A	G	Y	PTS: no antecedent event
34	50	M	R	A	G	Y	PTS: heavy lifting
35	74	M	R	A	G	Y	PTS: post-infection
36	30	M	R	A	G	Y	Inj: forearm
37	67	M	R	A	P 3,4,5	Y	PTS: no antecedent event
38	47	F	L	A	G	Y	PTS: post-botulinum toxin injection
39	65	M	L	A	P 3,4,5	Y	PTS: post-influenza vaccine
40	56	M	L	C	G	Y	Entrapment: Arcade of Frohse
41	58	M	L	C	P 3,4,5	N	C7 and C8 radiculopathy
42	58	M	R	A	G	Y	Inj: forearm
43	47	M	R	A	P 3,4,5	Y	PTS: no antecedent event
44	38	M	L	A	G	Y	Inj: forearm
45	48	F	L	C	P 1,2	Y	Idiopathic
46	71	M	L	A	G	Y	PTS
47	68	F	L	A	G	Y	PTS
48	53	M	L	U	G	Y	Idiopathic
49	17	M	L	U	G	Y	Idiopathic
50	43	M	L	A	G	Y	Idiopathic
51	63	F	R	U	P 3,4,5	N	Tendon rupture
52	31	M	L	A	G	Y	Iatro: biceps tendon repair
53	58	F	R	U	G	N	C7 and C8 radiculopathy
54	70	M		U	P 4,5	N	Tendon rupture
55	34	M	R	C	G	Y	Idiopathic
56	38	M	L	A	G	Y	Iatro: biceps tendon repair
57	77	F	L	A	G	Y	PTS: no antecedent event
58	75	M	L	U	G	Y	Idiopathic
59	53	M	R	A	G	Y	Iatro: hematoma after dialysis
60	63	M		A	P 1	N	Inj: muscle/tendon from cat bite
61	69	M	R	A	P 4,5	N	C7 and C8 radiculopathy
62	72	M	R	A	G	Y	Inj: forearm
63	72	M	L	C	G	Y	Idiopathic
64	59	M	L	A	G	Y	Inj: forearm
65	46	F	L	A	G	Y	PTS: no antecedent event
66	42	F	R	C	G	Y	Idiopathic
67	74	M	R	A	P 4,5	N	C7 and C8 radiculopathy
68	51	M	L	A	P 1,2,3,4	Y	Inj: muscle/tendon: dog bite
69	70	F	R	A	P 3,4,5	N	C7 and C8 radiculopathy
70	52	F	L	C	P 4,5	Y	Idiopathic
71	64	M	R	A	P 2,3	N	C7 and C8 radiculopathy
72	75	F	R	A	P 4,5	N	C7 and C8 radiculopathy
73	71	F	R	A	P 3,4	N	C7 and C8 radiculopathy
74	42	F	R	A	P 3	N	C7 and C8 radiculopathy
75	68	M	R	A	G	N	C7 and C8 radiculopathy
76	71	M	L	A	P 3,4,5	Y	PTS: no antecedent event
77	54	M	R	A	P 3,4,5	N	C7 and C8 radiculopathy
78	56	M	R	A	P 3,4,5	N	C7 and C8 radiculopathy
79	81	M	R	A	G	Y	Compression by cyst
80	68	F	R	A	G	Y	Inj: fracture of radius neck
81	51	M	R	A	G	Y	Iatro: surgery at antecubital fossa
82	52	F	L	A	P 1,2	Y	PTS: no antecedent event
83	59	M	R	A	G	N	PTS: no antecedent event
84	54	F	L	A	G	Y	Iatro: surgery for fracture of radius neck
85	63	M	R	A	G	Y	PTS: no antecedent event
86	55	F	L	A	P 3,4,5	N	C7 and C8 radiculopathy
87	72	F	R	A	P 4,5	N	Tendon rupture

**Figure 3:**
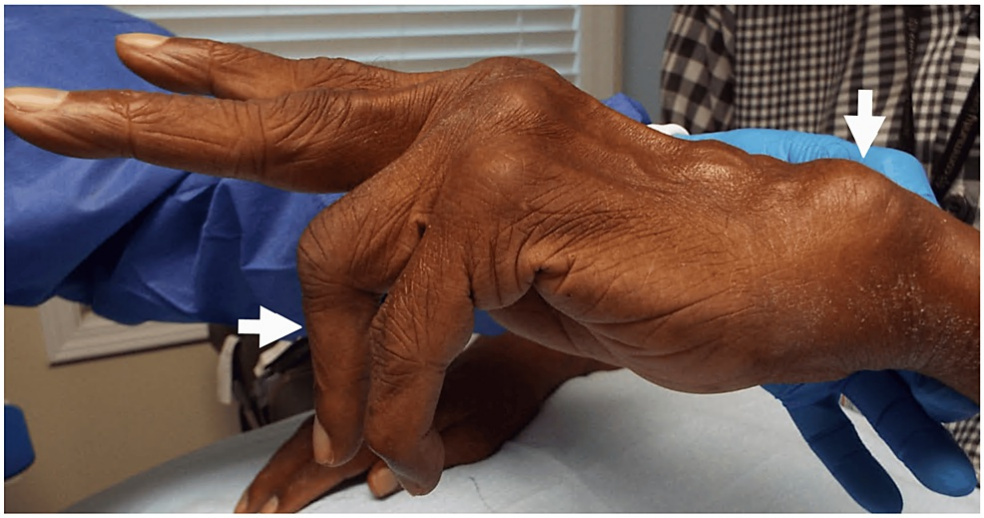
Partial Finger Drop Involving Digits 4 and 5 of the Left Hand Partial finger drop involving digits 4 and 5 of the left hand (horizontal arrow) caused by ruptured tendons in a patient with rheumatoid arthritis (Vaughan-Jackson syndrome). Note the swelling of joints and the dorsally subluxated ulnar head (vertical arrow).

**Figure 4:**
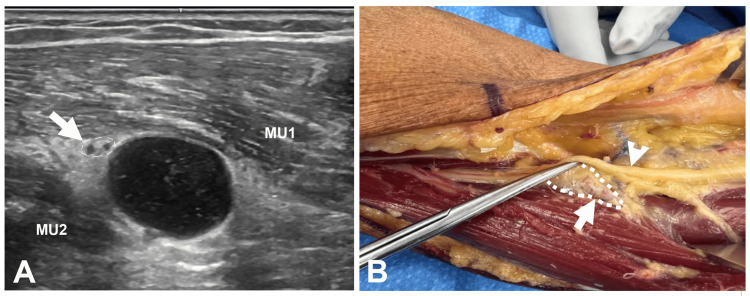
Finger Drop Caused by Compression by a Ganglion Cyst (A) Ultrasound: short axis view at the lateral elbow showing the PIN (arrow) with two fascicles (hypoechoic dots within a circle). MU1: extensor digitorum muscle; MU2: extensor carpi ulnaris muscle. (B) The patient underwent neurolysis of the radial nerve, excision of the ganglion cyst, and elbow arthrotomy. The intraoperative photo shows the cyst (oblique arrow, within a circle) and the overlying PIN (vertical arrowhead). PIN: posterior interosseous nerve

The most common cause of finger drop was PTS (29 [33%] patients), followed by either iatrogenic or other injuries (23 [26%]; iatrogenic: 13 patients, other: 10 patients), cervical radiculopathy (16 [18%] patients), idiopathic (13 [15%] patients), ruptured tendon (four [5%] patients) (Figure 3), and compression/entrapment (two [2%] patients; one from compression by a ganglion cyst (Figure 4) and the other from entrapment at the Arcade of Frohse), confirmed by surgery (Table 1). Of the 16 patients who experienced cervical radiculopathy as the cause of the finger drop, 15 patients had a C7 and C8 radiculopathy and one patient had a C7 radiculopathy.

**Figure 5:**
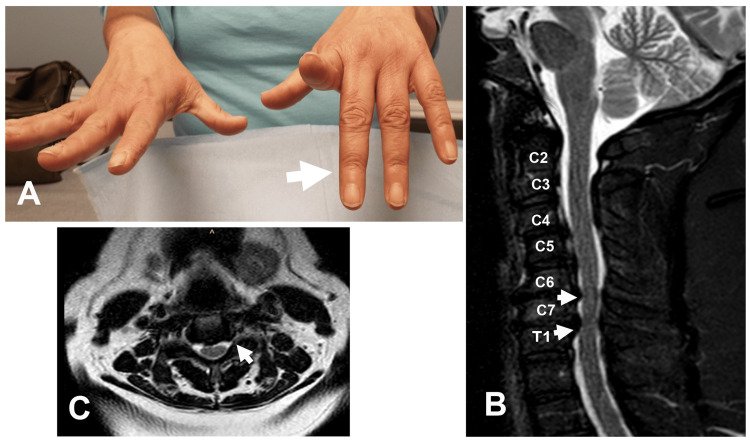
Partial Finger Drop Caused by Cervical Radiculopathy (A) Partial finger drop involving digits 3, 4, and 5 of the left hand (arrow). MRI of the (B) sagittal view showing cervical spondylosis at C6-7 and C7-T1 (arrows) and (C) axial view at C6-7 showing left foraminal stenosis at C6-7 (arrow).

Of the 87 patients who had clinical features of finger drop, 64 (74%) showed evidence of PIN involvement (Table 1). A total of 13/16 (81%) patients with cervical radiculopathy and all four patients with a tendon rupture experienced partial finger drop, while the majority (52/64 [81%]) of patients with PIN neuropathy developed global finger drop. Figure 5 shows a patient who presented with partial finger drop involving the ulnar three digits in whom the underlying cause was C7 and C8 radiculopathy.

Of the 29 patients with PTS, 17 (59%) had an antecedent event. These events included medical/surgical procedures in seven (41%), physical exertion in four (24%), vaccination in four (24%) (two following the COVID-19 vaccine, one after the botulinum toxin, and one following the influenza vaccine), and other events in two (12%) patients (one after sustaining a fall and the other after experiencing blunt injury). The left side (17 patients [59%]) was more commonly affected.

**Figure 6:**
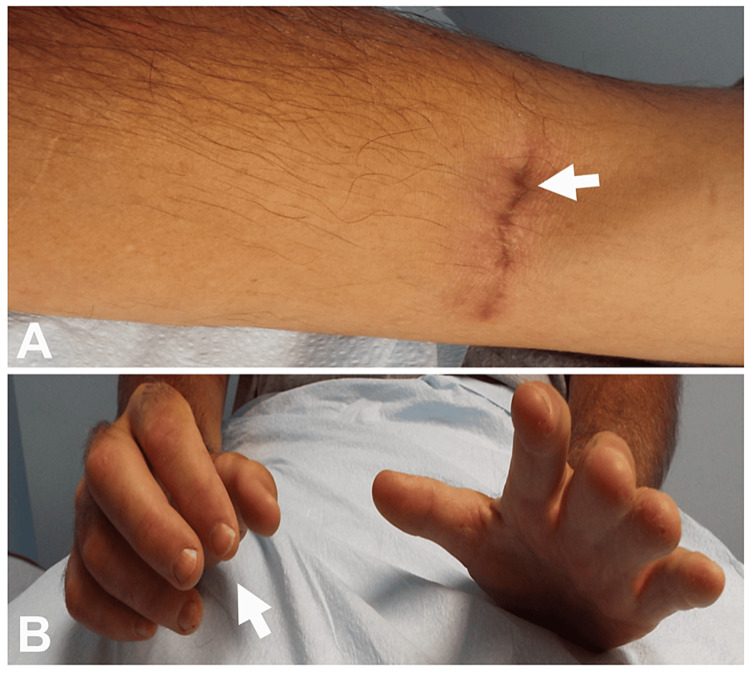
Global Finger Drop Caused by Iatrogenic Injury to Posterior Interosseous Nerve During Placement of AV Fistula for Dialysis (A) Iatrogenic PIN injury caused by the placement of AV shunt for dialysis (horizontal arrow points to the surgical scar). (B) Global finger drop on the right (oblique arrow) with intact dorsiflexion. PIN: posterior interosseous nerve; AV: arteriovenous

Of the 13 patients whose finger drop resulted from an iatrogenic injury, eight (62%) had left-side involvement (Table 2). A biceps tendon repair at the antecubital fossa was the most frequent surgical procedure antecedent to the onset of finger drop (six [46%] patients). Figure 6 shows a global finger drop in a patient who suffered an iatrogenic injury to PIN during the placement of an arteriovenous fistula for dialysis.

**Table 2:** Iatrogenic PIN Injuries Causing Finger Drop: EMG and Ultrasound Findings PIN: posterior interosseous nerve; EDX: electrodiagnostic findings; Ultrasound findings: 1. Increase in cross-sectional area of PIN (>5 mm^2^) 2. Enlarged fascicles 3. Hypoechoic nerve 4. Hyperechoic nerve 5. Neuroma in continuity

Patient Number	Side	Surgery	EDX Findings	Ultrasound
1	L	Excision lipoma forearm	PIN: Demyelination, Partial axon loss	1,3
2	L	Ligament of Struthers and pronator teres release	PIN: Partial axon loss	1,2
3	L	Biceps tendon repair	PIN: demyelination, Partial axon loss	1
4	R	Biceps tendon repair	PIN: Total axon loss	1,2
5	R	Biceps tendon repair	PIN: Partial axon loss	Not done
6	L	Biceps tendon repair	PIN: Conduction block	1
7	L	Biceps tendon repair	PIN Total axon loss	1,4
8	L	Biceps tendon repair	PIN: Total axon loss	1,2,3
9	R	Dialysis portal at antecubital fossa	PIN: Total axon loss	1,3
10	R	Dialysis portal at antecubital fossa	PIN: Conduction block	1,2
11	L	Repair of radial head fracture	PIN: Total axon loss	1,2,5
12	R	Multiple surgeries at elbow	Normal (muscle/tendon injury)	Not done
13	L	Fracture of radius neck	PIN: Total axon loss	1,3

EDX studies

Of the 29 patients with PTS, the PIN was the only nerve affected in 27 (93%) patients, while both the PIN and lower trunk of the brachial plexus were involved in one (3%) patient and only the lower trunk in one (3%) patient (Table 3). The EDX studies revealed partial axon loss in 18 (62%) patients, conduction block in eight (28%) patients, and total axon loss in four (14%) patients. The presence of denervation changes like fibrillations and positive waves in the paralyzed muscles indicate axon loss; in the conduction block, fibrillations and positive waves are not seen.

**Table 3:** Parsonage-Turner Syndrome Presenting as Finger Drop: EMG and Ultrasound Findings PIN: posterior interosseous nerve; EMG: electromyography Ultrasound: 1. Increase in cross-sectional area of PIN (>5 mm^2^) 2. Enlarged fascicles 3. Hypoechoic nerve 4. Hyperechoic nerve 5. Neuroma in continuity

Patient Number	Side (L/R)	Nerves Affected	EMG	Ultrasound
1	L	PIN	Total axon loss	1,2
2	R	PIN	Partial axon loss	Normal
3	L	PIN	Total axon loss	Normal
4	R	PIN	Conduction block	Normal
5	L	PIN	Partial axon loss	Normal
6	R	PIN	Partial axon loss, conduction block	Normal
7	L	PIN	Partial axon loss	Normal
8	L	PIN	Conduction block	Not done
9	R	PIN	Conduction block	Normal
10	L	PIN	Partial axon loss	Normal
11	R	PIN	Partial axon loss	Normal
12	L	PIN	Partial axon loss	Normal
13	L	PIN	Conduction block	Not done
14	R	PIN, lower trunk	Conduction block	Not done
15	L	PIN	Partial axon loss	Normal
16	R	PIN	Partial axon loss	2
17	R	PIN	Partial axon loss	Normal
18	R	PIN	Partial axon loss	2
19	L	PIN	Partial axon loss	Not done
20	L	PIN	Partial axon loss	Not done
21	R	PIN	Partial axon loss	2
22	L	PIN	Total axon loss	Not done
23	L	PIN	Partial axon loss	Not done
24	L	PIN	Partial axon loss	2
25	L	PIN	Conduction block	Not done
26	L	PIN	Conduction block	1
27	L	PIN	Partial axon loss	1
28	R	Lower trunk	Partial axon loss	Not done
29	R	PIN	Total axon loss	1,2

Of the 13 patients who sustained an iatrogenic injury prior to the finger drop, the PIN was involved in 12 (92%) patients (Table 2). The EDX studies demonstrated total axon loss in six (46%) patients, partial axon loss in four (31%), demyelination in two (15%) patients, and conduction block in two (15%) patients. One (8%) patient had a normal EDX study, and the finger drop was the result of muscle/tendon injuries resulting from multiple surgical procedures at the elbow and forearm.

US studies

Of the 20 patients with PTS who underwent US, eight (40%) showed enlarged fascicles in the PIN/distal part of the radial nerve. No typical hourglass constrictions were documented in the distal part of the radial nerve or in the PIN. The US was normal in 12 (60%) patients.

Of the 11 patients with iatrogenic injury to the PIN and underwent US, enlarged fascicles were noted in all. The nerve appeared hyperechoic in three (27%) patients and hypoechoic in another three (27%). A neuroma in continuity was noted in one (9%) patient.

US detected a ganglion cyst causing compression of the PIN in one patient. Figure 4 shows the US and intraoperative pictures; the cyst was found to be communicating with the radiocapitellar joint.

## Discussion

A thorough neurological examination is the most important first step in recognizing and ascertaining the cause of finger drop. Determining whether a patient has a partial or global finger drop can provide clues regarding the underlying etiology. In our series, 81% of patients with cervical radiculopathy and all patients with tendon injury/rupture had partial finger drop, while 88% of patients with PIN involvement had global finger drop. EDX studies serve as an important next step in the further evaluation of patients presenting with finger drop; EDX findings can differentiate between finger drop due to nerve involvement from finger drop secondary to tendon injury/rupture. They can also provide insight into the severity of nerve injury by distinguishing between conduction block and axon loss. Additionally, serial EDX studies can monitor the return of nerve function by the presence of reinnervation changes in muscles and improvement in nerve conduction. MRI and US are useful in identifying muscle and/or tendon injuries as well as tendon ruptures, which may be the underlying cause of the finger drop in some patients [14]. US study is cheaper than MRI and has the advantage of being readily available. It can detect neurotmesis, neuroma in continuity, and lesions that may be compressing/entrapping the PIN.

PIN neuropathy was the most common cause of finger drop in our study, comprising 74% of the patients. At the radiocapitellar joint of the elbow, the radial nerve bifurcates into the PIN and the superficial radial nerve after which the PIN passes between the two heads of the supinator muscle and wraps around the radial neck [16]. As it emerges from the supinator muscle, the PIN separates into numerous divisions supplying the group of muscles of the dorsal forearm which extend to the digits at the metacarpophalangeal joints. The PIN is most vulnerable to entrapment just beyond its origin as it passes through the fibers of the supinator muscle in the proximal forearm [5]. The PIN may be entrapped at the arcade of Frohse or compressed by space-occupying lesions (lipomas, ganglion cysts, and rheumatoid synovial overgrowth), or may experience fascicular dysfunction due to radial nerve injuries [5,16]. Figure 4 shows one of the patients in this series with global finger drop, in whom a cyst was detected compressing the PIN by US study.

Depending on the location of the PIN pathology, all (global) or only certain fingers may show finger drop (“Texas longhorn” hand gesture due to partial paralysis of finger extensors) [16]. Clinical distinction between partial finger drop due to cervical radiculopathy versus partial injury to the PIN (neurogenic finger drop) and those resulting from tendon injury/rupture (non-neurogenic finger drop) may be difficult without needle EMG study of the extensor muscles. In this series there were six patients with non-neurogenic finger drop confirmed by normal conduction in the PIN along with the absence of denervation changes in the extensor muscles; two of the cases presented with finger drop affecting the small and ring fingers due to tendon rupture secondary to subluxation of the ulnar head related to rheumatoid arthritis (Figure 3); in these patients, initially the small finger dropped and later the ring finger, typical of Vaughan-Jackson syndrome (Figure 3) [17].

The appropriate treatment for finger drop depends on the underlying cause of the extensor weakness. Conservative therapy includes rest, non-steroidal anti-inflammatory medications, and/or steroid injections [5]. Finger drop due to cervical radiculopathy warrants prompt surgical intervention to improve patient outcomes [9]. Additionally, surgical exploration of the PIN and its branches may be necessary in cases where entrapment or compression of the PIN is the source of the finger drop. In patients with finger drop from PIN neuropathy who have no detectable cause, there is a higher likelihood of restoration of muscle function if an interfascicular neurolysis and tendon transfer are performed within seven months of the injury [18]. The discovery of hourglass constriction in the fascicles of the radial nerve that contribute to the PIN in patients presenting with features of PTS has led to the consideration of surgical options such as microneurolysis with the potential for recovery [19,20].

Strengths and limitations

The strength of our study is the large cohort of patients with finger drop and the use of both EDX studies and US in their evaluation. We highlighted the multiple causes of finger drop and determined whether partial or global finger drop were more likely with specific causes of finger drop. Limitations of our study include its retrospective nature and lack of follow-up of many of the patients after their EDX evaluation which has limited our ability to assess the long-term outcome.

## Conclusions

PIN neuropathy is the most frequent cause of finger drop, however, other conditions such as cervical radiculopathy and extensor tendon rupture may also be responsible. Global finger drop is suggestive of PIN neuropathy, while partial finger drop occurs more often in cervical radiculopathy and tendon rupture. EDX and US studies should be used as complementary tests to distinguish between neural and non-neural causes and to determine the location and the etiology of finger drop so that appropriate treatment can be formulated. Physicians should be cognizant of the various etiologies of finger drop and be aware of the different finger drop patterns that may provide useful clues to the underlying etiology.
